# Role of Metal Ions and Hydrogen Bond Acceptors in the Tautomeric Equilibrium of Nitro-9[(Alkylamino)Amino]-Acridine Drugs

**DOI:** 10.1155/S156536330400007X

**Published:** 2004

**Authors:** Maria Carlone, Nicola G. Di Masi, Luciana Maresca, Nicola Margiotta, Giovanni Natile

**Affiliations:** Dipartimento Farmaco-Chimico Università di Bari via E. Orabona, 4 Bari I-70125 Italy

## Abstract

3-nitro-9-[2-(dialkylamino)ethyl)]aminoacridines (alkyl = methyl or ethyl) have been used as ligands towards platinum(If). The end product is a complex in which the acridine acts as a tridentate ligand contributing the two exocyclic nitrogen atoms and one of the two peri carbons. The metallation takes place predominantly at the peri position of the unsubstituted ring. The coordinated acridine is in the imino tautomeric form although, in the free state, it occurs exclusively in the amino form (both in the solid state and in solution). The imino tautomer is considered to be the biologically active form. In the platinated species the N(10)H of the acridine can be involved in strong hydrogen bonding with a chloride ion leading to formation of an association complex, the formation constant has been found to be 1.4±10^3^ M^−1^. The N(10)H┄CI interaction can influence the tautomeric equilibrium of the acridine dye also in the uncoordinated species, however, the shift in favor of the imino tautomer is not complete.


### Role of Metal Ions and Hydrogen Bond Acceptors in the Tautomeric Equilibrium of Nitro-9[(Alkylamino)Amino]-Acridine Drugs.

Maria Carlone, Nicola G. Di Masi, Luciana Maresca,* Nicola Margiotta, and Giovanni Natile

*Dipartimento Farmaco-Chimico, Università di Bari, via E. Orabona, 4, I-70125 Bari (Italy)*

*mailto: maresca@farmchim.uniba.it*

### ABSTRACT

3-nitro-9-[2-(dialkylamino)ethyl)]aminoacridines (alkyl = methyl or ethyl) have been used as ligands towards platinum(II). The end product is a complex in which the acridine acts as a tridentate ligand contributing the two exocyclic nitrogen atoms and one of the two peri carbons. The metallation takes place predominantly at the peri position of the unsubstituted ring. The coordinated acridine is in the imino tautomeric form although, in the free state, it occurs exclusively in the amino form (both in the solid state and in solution). The imino tautomer is considered to be the biologically active form. In the platinated species the N(10)H of the acridine can be involved in strong hydrogen bonding with a chloride ion leading to formation of an association complex, the formation constant has been found to be $1.4 \pm 10^3$ $M^{-1}$. The N(10)H⋯Cl interaction can influence the tautomeric equilibrium of the acridine dye also in the uncoordinated species, however, the shift in favor of the imino tautomer is not complete.

### INTRODUCTION

Acridines are interesting molecules well known for their DNA intercalating abilities and for the pharmacological properties they can develop in the presence of appropriate substituents.

In particular, nitracrine-WHO (named also ledakrin) has been used as an antineoplastic drug /1,2/, while quinacrine, a former antimalaric agent effective for the treatment of lupus erythematosus and giardiasis /3,4/, appears to be a candidate for the treatment of Creutzfeld-Jacob disease /5/. Moreover EPR and laser flash photolysis experiments have shown that photo-excited quinacrine is a producer of superoxide and hydroperoxy adducts and that quinacrine photoproducts produce singlet oxygen, rendering the molecule a candidate for photodynamic therapy /6/.

Both the above quoted acridines share the presence of a diamino alkyl chain stemming from C(9).

The presence and the position of substituents in the acridine ring system are also crucial for the biological activity of these compounds. Isomers of nitracrine-WHO, having the nitro group in ring positions other than 1, completely lose the antitumor activity. The efficacy is also related to the number of methylene spacers

**nitracrine-WHO**

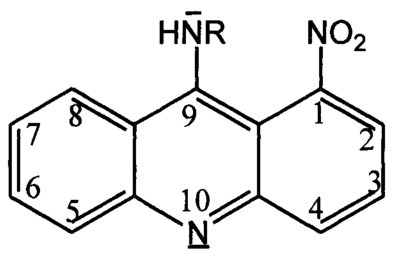

R = $(CH_2)_3NMe_2$

**quinacrine**

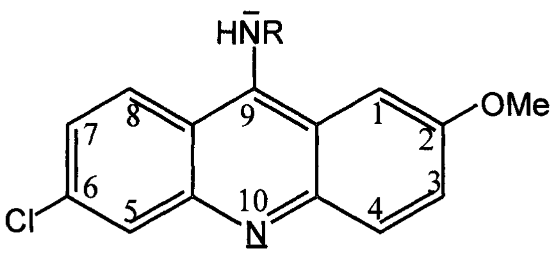

R = $CHMe(CH_2)_3NEt_2$

between the two aminic nitrogens of the aliphatic side chain (the highest activity being observed for 2 or 3 methylene spacers) /7/.

All 9-aminoacridines having at least a hydrogen atom on the nitrogen bound to C(9) can exhibit an amino-imino tautomerism that has attracted particular attention in recent years. Several spectroscopic and theoretical investigations, concerned with this particular aspect of the acridine behavior, have been reported /8-10/. The position of the tautomeric equilibrium appears to be influenced by the presence and position of substituents, and also by the nature of the solvent since the two tautomers have significantly different polarity /11/.

In the case of nitro-9-(aminoalkyl)aminoacridines the 2- and 3-nitro isomers are present, both in solution and in the solid state, in the planar amino form; in contrast the 1-nitro isomer is present as pure imino tautomer in the solid state and as a mixture of amino and imino forms in solvents of high polarity /11,12/.

As already anticipated the presence of the imino tautomer appears to be relevant to the biological activity of these molecules. Apart from the antitumor activity of the 1-nitro isomers, the 1- and 4-nitro isomers show hypoxia selective cytotoxicity (similarly to 1-nitro species, also the 4-nitro isomers are present in solution as a mixture of the two tautomers) /8/.

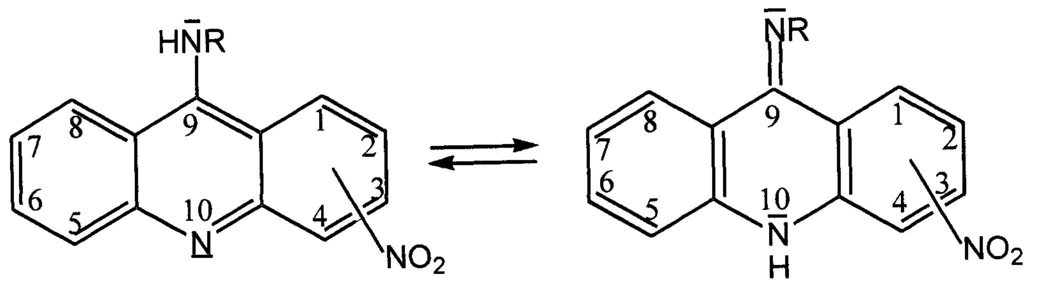

By using these molecules as ligands towards platinum(II) it could be possible to obtain bifunctional drugs combining alkylating abilities (driven by the metal) and intercalating properties (driven by the acridine) /13-15/.

The results have not always been encouraging since loading with a metal can enhance chemical toxicity /16/; however, very recently a platinum-acridine conjugate, which is very active towards HL-60 leukaemia cells, has been reported (the unbound acridine moiety has a much lower efficacy against the same cell line) /17/.

In a previous study we investigated the kinetically controlled products in the reaction of different platinum(II) substrates with the biologically active 1-nitro-9-[(2-dialkylaminoethyl)amino]acridines (alkyl = methyl or ethyl; $L_1$ and $L_2$, respectively) and the inactive 3-nitro isomers ($L_3$ and $L_4$; for alkyl = methyl and ethyl, respectively). The kinetic products were found to be dependent upon the charge of the metal substrate and the position of the tautomeric equilibrium in the free acridine. Anionic and neutral platinum species reacted with the amino and the imino tautomers to give the endocyclic N(10) and the exocyclic C(9)N-bonded derivatives, respectively (the contemporary presence of the two forms, in the free acridine, generated a mixture of the two products). In contrast, positively charged substrates coordinate exclusively to the exocyclic aminic nitrogens and stabilize the imino form also in the case of 3-nitro acridines which exist as pure amino tautomers in the free state (used solvents: chloroform and acetone) /18/.

There is only one report concerning a 9-amino acridine in which platinum coordination through C(9)N (exocyclic nitrogen) and amino form of the ligand were proposed. The proposed stereochemistry was based on the downfield shift of two aromatic signals assigned to H(1) and H(8) protons /19/. Their data, however, are also in accord with an acridine coordinated through N(10) (endocyclic nitrogen) and the downfield shifted resonances belonging to H(4) and H(5). Being the complex neutral (*cis*-[$Pt(C_6H_5)_2$(CO)(9-Aminoacridine)]) the involvement of the endocyclic nitrogen is not surprising /18/, while the proposed situation (involvement of the exocyclic nitrogen and persistence of the amino form) is very unusual.

In a second study we investigated the thermodynamically controlled reaction products in the case of 1-nitro-9-[(2-dialkylaminoethyl)amino]acridines ($L_1$ and $L_2$). In the end products the acridine acted as a tridentate ligand contributing the two exocyclic nitrogens and the peri carbon C(8). The ligand was in the imino form and the N(10)H showed a strong tendency to be engaged in hydrogen bonding both in the solid state and in solution /20/.

We have now extended the investigation to the thermodynamically controlled products in the reaction of platinum substrates with 3-nitro-9-[(2-dialkylaminoethyl)amino]acridines ($L_3$ and $L_4$) and analyzed the strength of N(10)H bonding interaction. The results are reported hereafter.

### EXPERIMENTAL

### Starting Materials

The complex *cis*-[$PtCl_2(DMSO)_2$] was prepared according to reference 21. Dry chlorinated solvents were obtained by distillation from calcium hydride. 3-Nitro-9-[(2-dimethylaminoethyl)amino]acridine, $L_3$, and 3-Nitro-9-[(2-diethylaminoethyl)amino]acridine, $L_4$, were kindly supplied as dihydrochlorides by J. Konopa (Gdansk Polytechnic, Poland). The dihydrochlorides (0.2 mmol in a typical experiment) were suspended in dry dichloromethane (8 mL) and treated with the stoichiometric amount of powdered KOH (0.4 mmol). After a brief stirring the deep red solution was filtered under argon atmosphere and evaporated to dryness to afford the free acridine.

### Preparation of Complexes

Complexes [PtCl(L-H)] were prepared by reaction of *cis*-[$PtCl_2(DMSO)_2$] with the appropriate ligand in dry $CH_2Cl_2$. In a typical experiment 1 mmol of both reagents and 10 mL of solvent were used and the mixture was refluxed for 4 hours in argon atmosphere. While the reaction was progressing, [$PtCl_2$ $(DMSO)_2$] dissolved and the red color deepened. An excess of LiCl (2-3 mmol) was then added and the mixture was kept under stirring for two more hours (the need for this step is to eliminate possible cationic species still having a DMSO molecule in the coordination sphere of platinum). The reaction solution was concentrated to small volume and then chromatographed over a silica gel column. The first yellow fraction, eluted with $CH_2Cl_2$, contained a few mg of the product of hydrolysis of the acridine ligand to the corresponding 9-acridone. The main fraction was eluted with dichloromethane containing 3% (v/v) of acetone; evaporation to dryness under reduced pressure gave a dark red solid of formula [PtCl(L-H)] (elemental analyses) which contained always some co-crystallized molecules of acetone. Small amounts of other products could be eluted using higher contents of acetone (> 10 %, v/v) but they have not been identified at this stage of the work.

[PtCl($L_3$-H)], **1**. The yield, referred to platinum, was 60%. Calcd for $C_{17}H_{17}ClN_4O_2Pt$ $(CH_3)_2CO$: C, 40.17; H, 3.88; N, 9.37%. Found: C, 39.53; H, 3.77; N, 9.22%. A second chromatographic run on the main product, eluted with dichloromethane containing 3% (v/v) of acetone, showed the presence of a second species having slightly lower retention time and formed in very low yield. This compound had identical elemental analyses and has proven to be an isomeric species, differing in the metallation site: C(1) instead of C(8). The yield of the C(1) metallated product was only ~5% of the total metallated compound. The two isomers will be indicated as **1a** and **1b**.

[PtCl($L_4$-H)], **2** . The yield, referred to platinum, was 55 %. Calcd for $C_{19}H_{21}ClN_4O_2Pt$ $(CH_3)_2CO$: C, 42.21.; H, 4.35; N, 8.95%. Found: C, 41.70; H, 4.20; N, 9.00%.

### Physical Measurements

Proton NMR spectra were recorded on a Bruker AM 300 spectrometer.

### Evaluation of the association constants.

A 0.8 mL solution (1.4 mM) of the metallated platinum compound, placed in a NMR tube, was treated with aliquots of tetrabutylammonium chloride and the $^1H$ NMR spectrum recorded after each addition. A titration curve was generated by plotting the changes in N(10)H chemical shift versus the concentration of chloride ions. Since **1a** always co-crystallizes with some acetone, the N(10)H chemical shift, at zero acetone content, was evaluated by extrapolation to zero of a quasi linear plot reporting the chemical shift of N(10)H against the content of acetone. The association constants were determined by a non-linear regression analysis using the computer program Wiener, kindly made available by Prof. M. J. Hynes, National University of Ireland, and Galway, Ireland /22/.

### RESULTS AND DISCUSSION

### Coordination to platinum of 3-nitro-9- [(2-dialkylaminoethyl) amino]acridines

The reaction has been performed, as in the case of the 1-nitro isomers, using *cis*-$[PtCl_2(DMSO)_2]$ as platinum precursor. The reaction resulted in the formation of a major product, **1a**, analogous to that obtained in the case of the corresponding reaction with 1-nitro acridines.

Therefore in **1a** the acridine acts as a tridentate *NNC* ligand contributing the two exocyclic nitrogens and the peri carbon C(8); a chloride ligand completes the metal coordination sphere. The formulation is well supported by $^{1}H$ NMR data (Table 1). There are two sets of aromatic protons, each set containing three signals. In one set one signal is coupled with $^{195}Pt$ (Figure 1b) and can be assigned to H(7), the proton adjacent to the metallated carbon. The second set of three signals can be assigned to the protons of the ring bearing the nitro substituent. Both methylenes of the chelate ring show up as triplets, indicating that there is no hydrogen on the proximal nitrogen and therefore the ligand is present as imino tautomer and the signal close to 8 ppm ($CDCl_3$) is to be assigned to the protonated endocyclic nitrogen (N(10)H). Moreover the $^{3}J_{Pt,H}$ ($\approx$ 35 Hz) of one methylene is significantly higher than that of the other ($\approx$ 10 Hz) in accord with a different trans influence of the trans ligands (a chloride and a $\sigma$-carbon). The shift from the amino to the imino form also brings changes in the relative positions of some aromatic protons (Figure 2); in particular an up field shift is experienced by H(4) and H(5).

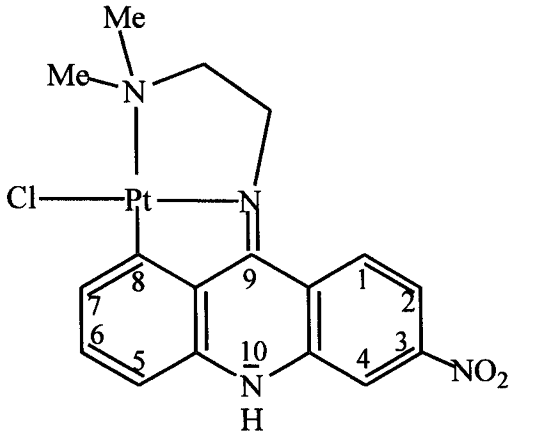

**1a**

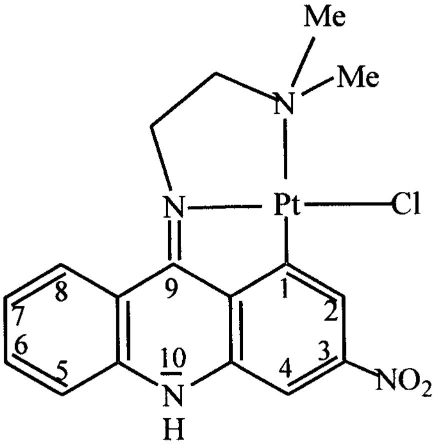

**1b**

We also had evidence of the formation of a second species, the C(1) metallated complex, **1b**. The $^{1}H$ NMR spectrum of this species, in the aliphatic portion, is very similar to that of the previous isomer. In contrast in the aromatic region it shows two sets of interconnected signals, one set comprises two resonances and the other set four resonances. One signal of the set of two also shows coupling with $^{195}Pt$ (Figure 2). It is clear that in **1b** metallation has occurred at the nitro-substituted ring (carbon C(1)); therefore the set of two resonances is to be assigned to protons H(2) (signal coupled with $^{195}Pt$) and H(4). On the other hand, the set of four resonances is to be assigned to protons H(5)-H(8) of the unsubstituted ring. Also for **1b** there is a

**Table 1**

$^{1}H$ Chemical Shifts [ppm, downfield from $Si(CH_3)_4$]

| Compound | Solvent | H(1) | H(2) | H(4) | H(5) | H(6) | H(7) | H(8) | C(9)NH | N(10)H | $C_{prox}H_2$ | $C_{dist}H_2$ | R | |
|---|---|---|---|---|---|---|---|---|---|---|---|---|---|---|
| | | | | | | | | | | | | | $CH_3$ | $CH_2CH_3$ |
| **$L_3$** | $CDCl_3$ | 8.28 | 7.99 | 8.93 | 8.06 | 7.73 | 7.43 | 8.15 | | | 3.92 | 2.63 | 2.40 | |
| | $(CD_3)_2CO$ | 8.56 | 7.99 | 8.71 | 7.99 | 7.75 | 7.47 | 8.32 | | | 4.05 | 2.73 | 2.34 | |
| **[PtCl($L_3$-H)], 1a** | $CDCl_3$ | 8.34 | 7.84 | 8.16 | 6.73 | 7.34 | 7.48 | | | 8.18 | 4.47 | 3.30 | 2.93 | |
| | $(CD_3)_2CO$ | 8.51 | 7.81 | 8.24 | 6.74 | 7.31 | 7.21 | | | 10.51 | 4.45 | 3.40 | 2.92 | |
| **[PtCl($L_3$-H)], 1b** | $(CD_3)_2CO$ | | 7.99 | 7.62 | 7.49 | 7.72 | 7.24 | 8.4 | | 10.47 | 4.60 | 3.39 | 2.90 | |
| **$L_4$** | $CDCl_3$ | 8.28 | 7.98 | 8.93 | 8.07 | 7.72 | 7.42 | 8.16 | 7.16 | | 3.92 | 2.80 | | 2.68 ($CH_2$)<br>1.12($CH_3$) |
| **[PtCl($L_4$-H)], 1a** | $CDCl_3$ | 8.35 | 7.84 | 8.15 | 6.71 | 7.34 | 7.54 | | | 8,18 | 4.51 | 3.34 | | 3.32; 3.16 ($CH_2$)<br>1.41 ($CH_3$) |

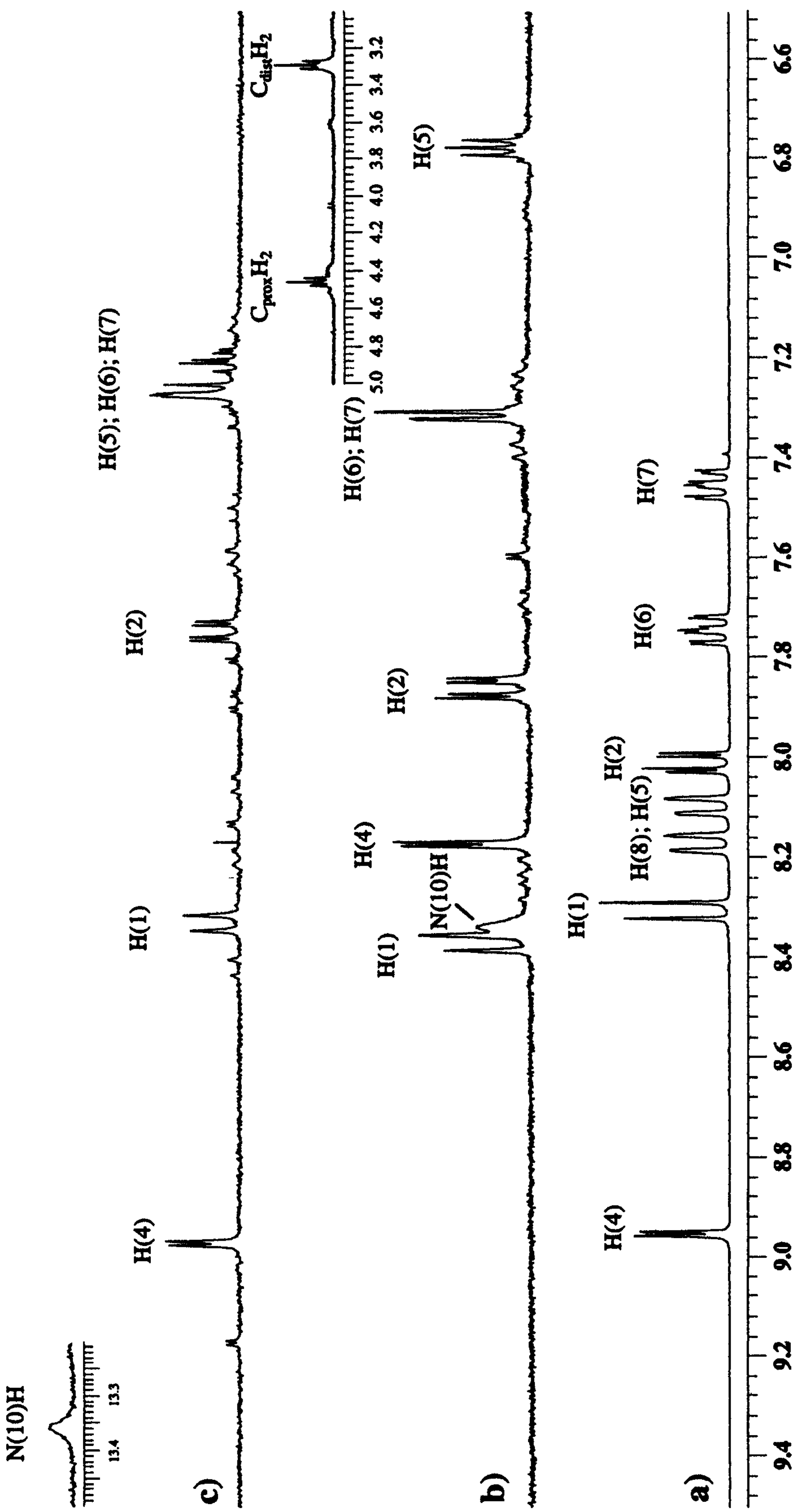

**Fig. 1:** $^1$H NMR spectrum ($CD_2Cl_2$, region of aromatic protons) of: a) ligand $L_3$; b) complex [PtCl($L_3$-H)], **1a**, with an insert showing methylene protons; c) complex [PtCl($L_3$-H)] in the presence of chloride ions, with an insert showing N(10)H.

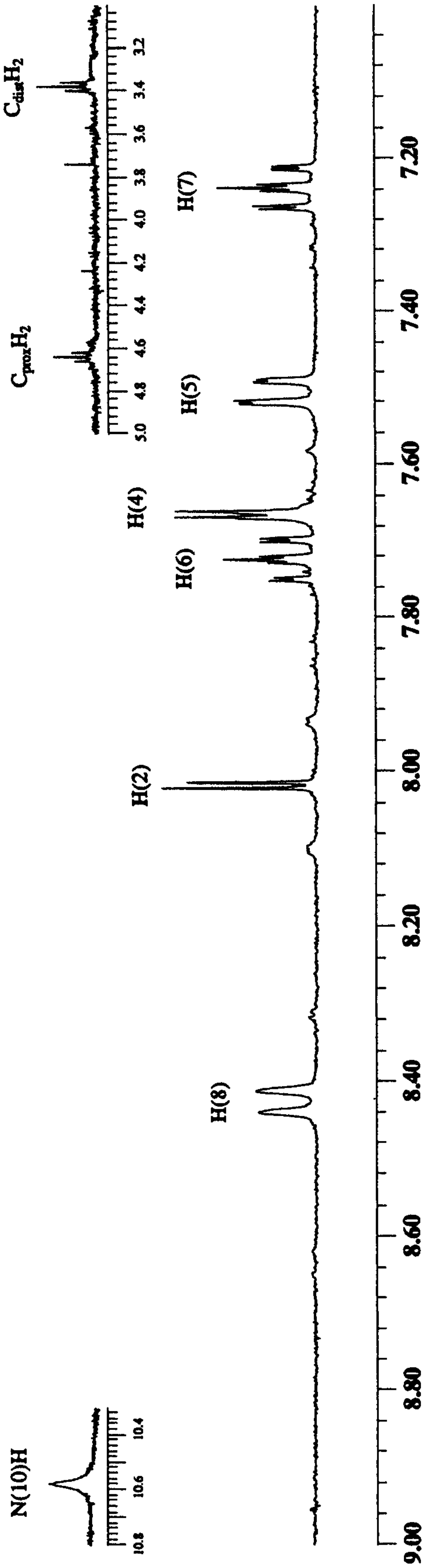

**Fig. 2:** $^1$H NMR spectrum (acetone-$d_6$, region of aromatic protons) of complex [PtCl($L_3$-H)], **1b**, the inserts show N(10)H and methylene protons.

upfield shift of H(4) and H(5) and a resonance at 10.50 ppm (acetone-$d_6$) arising from N(10)H. The very small yield of compound **1b** can be rationalized on the basis that the metallation reaction can be considered an electrophilic attack of the metal on the carbon atom and between C(8) and C(1). C(1) is a much weaker nucleophile due to the presence in the ring of an electron withdrawing substituent such as the nitro group.

### Platinum-acridine complexes as hydrogen-bond donors

In the [PtCl(L-H)] species isolated in the present study, as in the analogous species previously obtained starting from 1-nitroacridines, N(10)H exhibits a marked tendency to be involved in a hydrogen bond. In solution the N(10)H resonance of the metallated complexes undergoes a remarkable downfield shift if chloride ion is added. The maximum $\Delta\delta$ is close to 5 ppm in chlorinated solvents and to 3.5 ppm in deuteroacetone. The low field value is quite the same in both solvents, but the starting value of N(10)H is *ca.* 1.5 ppm at lower field in the case of acetone than in the case of chlorinated solvents. Most likely acetone itself acts as a hydrogen bond acceptor which explains the deshielding observed in this solvent, as compared to chlorinated solvents, in the absence of added chloride. The interaction of N(10)H with chloride ions also causes a significant downfield shift of the peri protons H(4) and H(5) ($\Delta\delta$ of *ca.* 0.6 ppm).

The interaction between N(10)H and a chloride corresponds to the formation of a true association complex for which the formation constant, $K_f$, can be evaluated by titration experiments. In defect of added chloride, only a fraction of the platinum complex will be associated with chloride ions; however, only one N(10)H signal is always observed indicating that the rate of exchange of chloride between different molecules of platinum substrates is fast in the NMR time scale and the observed N(10)H chemical shift is a concentration weighed average of its value in free and chloride-associated species. An example of chloride binding curve is given in Figure 3. The formation constants, determined by a non-linear regression analysis, were found to be $1.4\pm0.4\times10^3$ $M^{-1}$ in the present complex [PtCl($L_3$-H)], **1a**, and $4.6\pm0.4\times10^3$ $M^{-1}$ in the analogous complex with 1-nitroacridine [PtCl($L_1$-H)].

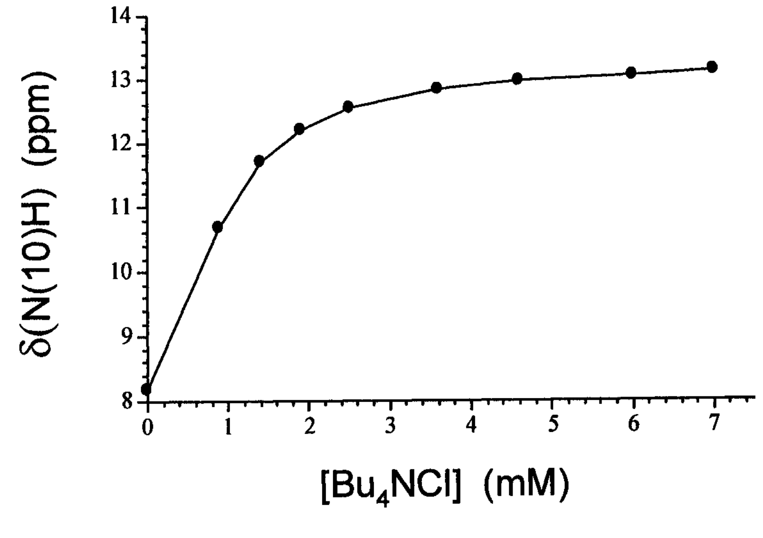

**Fig. 3:** Chloride titration curve for the complex [PtCl($L_1$-H)].

Also addition of bromide ions produces a remarkable downfield shift of the N(10)H proton, indicating the establishment of an association equilibrium similar to that observed in the case of chloride ion. The system, however, is complicated by the simultaneous partial substitution of bromide for chloride in the metal coordination sphere and was not further investigated.

Molecular association through hydrogen bonding, which can range from binary complexes to 2D and 3D networks, is a topic of wide interest. Among the systems more closely related to the one here studied, one should mention the cationic complexes of platinum(II) with bis(2-pyridyl)amine (dpa), $[PtMe(dpa)(Me_2SO)]^+$, reported by Romeo *et al.*, where NH of coordinated dpa strongly interacts with several counter anions (**A**, in the scheme) /23/.

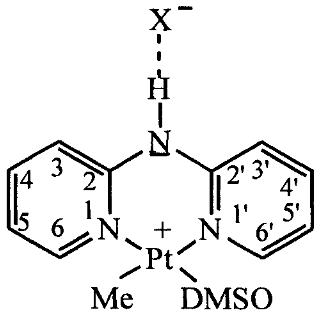

**A**

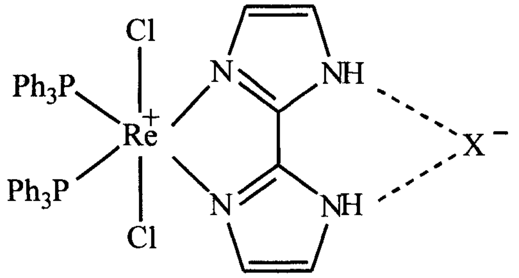

**B**

The link also persists in solution, as well evidenced by spectroscopic measurements. Of the examined anions, chloride was found to be the strongest H-bond acceptor. The pyridyl protons H(3) and H(3') of dpa were also shifted downfield as a consequence of the chloride association (there is a strict similarity between protons 3 and 3' of dpa and protons 4 and 5 of the acridine system.

A similar situation is offered by the NH groups of a coordinated biimidazole (biim) in some cationic complexes of rhenium(III), *e.g.* *trans*-$[ReX_2(PPh_3)(biim)]X$ (X = Cl, Br, I); also in this case tight ion pairs are formed in solution (**B**, in the scheme) /24/.

In a study on hallucinogenic receptor models, the interaction between the imidazolium chloride and some amphetamine analogs was investigated. The formation of a ternary complex between the phenethyl ammonium residue and the imidazolium cation, through the intermediacy of a chloride ion, was envisaged, and the formation constant, $K_f$, was calculated to be in the range 0.6 - 2 depending upon the type and the position of subsituents in the ring of phenethylamine /25/.

Finally, in the work by Green and Martin on complex formation between threehalomethanes and halide ions, the values of $K_f$ did not exceed 2.5 in $CCl_4$ ($CHCl_3/Cl^-$) and 3.8 in $CH_3CN$ ($CHI_3/Br^-$) /26/.

In view of the quoted examples, the association between the platinum complexes with the acridines (the acridine in its imino form) and chloride ions appears to be remarkably strong. So far similar equilibrium constants have only been observed in systems consisting of an anion interacting with several NH groups of a host /27/. Therefore it is very likely that in the acridine cases, too, the binding motif is not restricted to the interaction between the halide anion and N(10)H but also the adjacent protons C(4)H and C(5)H get involved in this type of interaction. This hypothesis is supported by the relevant downfield shift experienced by these CH protons upon addition of the halide anion.

**Influence of chloride ions on the tautomeric equilibrium in the free bases**

The effect of chloride ions on the tautomeric equilibrium of uncomplexed nitro-9-(aminoalkyl)aminoacridines was also investigated. In deutero acetone solution the two tautomeric forms of 1-nitro acridines are present in equilibrium and in rather low exchange rate with respect to the NMR time scale. The signals of the aromatic protons, particularly those of the unsubstituted ring which are significantly different for the two tautomers, are very broad, while the signal of the proton exchanging between N(10) and C(9)N was not detected; a considerable broadening also affects the resonance of the proximal methylene of the diaminic chain. Addition to these solutions of tetrabutylammonium chloride ($Cl^-$/acridine slightly above 1) causes a sharpening of all signals (the proximal methylene protons give rise to a well designed triplet) and the appearance of a resonance at 12.30 ppm assignable to N(10)H···Cl type of proton. The molecule is, however, present almost exclusively in its imino form, which has been stabilized *via* chloride association to N(10)H.

In the case of 3-nitro acridines, which in acetone solution are present as pure amino tautomers, the addition of chloride ions promotes a broadening of the NMR signals, which can be reasonably interpreted as slow exchange between different forms (very seemingly amino and imino tautomers).

In conclusion, a hydrogen bond acceptor, able to interact with N(10)H, can stabilize the imino form of a free acridine. However, much more efficient in the stabilization of the imino tautomer is the coordination to a metal ion of the exocyclic proximal nitrogen. Coordination to the metal ion increases the acidity of the exocyclic nitrogen with the consequence that the proton migrates on the endocyclic nitrogen N(10).

Finally metallation at a peri carbon atom further stabilizes the imino tautomer. In this case the transition to the imino tautomer is also favored by a bending of the acridine plane, with loss of aromaticity at the central ring, promoted by steric strain within a quasi planar five-ring system (two metal chelate rings and the three condensed rings of the acridine).

### ACKNOWLEDGEMENTS

This work was supported by the University of Bari, MIUR (COFIN N. 2001053898), and EC (COST Chemistry project D 20/WG0012-02). The authors are grateful to Prof. J. Konopa (Polytechnic of Gdansk, Poland) for the gift of acridine samples, and to Prof. M. J. Hynes, National University of Ireland, Galway, Ireland, for making available the computer program WinEQNMR.

### REFERENCES

1. C. Radzikowski, A. Ledochowski, M. Hrabowska, B. Horowska, B. Stefanska, J. Konopa and E. Morowska, *Arch, Immunol. Ther. Exp.*, **17**, 99 (1969).
2. A. Ledochowski and B. Stefanska, *Rocz. Chem.*, **14**, 3 (1966).
3. L. T. Webster Jr. *The Pharmacological Bases of Therapeutics*, 8th ed.; Pergamon: New York, 1990; Chapter 42, p 1005.

4. D. J. Wallace, *Sem. Arthritis Rheum.*, **18**, 282 (1989).
5. C. Korth, B. C. H. May, F. E. Cohen and S. B. Prusiner, *PNAS*, **98**, 9836 (2001).
6. A. G. Motten, L. J. Martinez, N. Holt, R. H. Sik, K. Reszka, C. F. Chignell, H.H. Tonnesen and J. E. Roberts, *Photochem. Photobiol.*, **69**, 282 (1999).
7. J. M. Woynarowski, A. Bartoszek and J. Konopa. *J. Chem. Biol. Interact.*, **49**, 311 (1984).
8. M. Boyd and W. A. Denny. *J. Med. Chem.*, **33**, 2656 (1990).
9. J. Rak, P. Skurski, M. Gutowski, L. Jozwiak and J. Blazejowski, *J. Phys. Chem.*, **101**, 283 (1997).
10. J. Rak, P. Skurski, M. Gutowski, L. Jozwiak and J. Blazejowski, *Austr. J. Chem.*, **101**, 283 (1997).
11. A. Tempczyk, J. Rak and J. Blâzejowski, *J. Chem. Soc. Dalton Trans.* **1990**, 1501.
12. J. J. Stezowski, P. Kollat, M. Bogucka-Ledokowska and J. P. Glusker, *J. Am. Chem. Soc.*, **107**, 2067 (1985).
13. B. E. Bowler, L. S. Hollis and S. J. Lippard, *J. Am. Chem. Soc.*, **106**, 6102 (1984).
14. B. E. Bowler and S. J. Lippard, *J. Biochemistry*, **25**, 3031 (1986).
15. B. E. Bowler, K. J. Amhed, W. I. Sundquist, L. S. Hollis, E. E. Whang and S. J. Lippard., *J. Am. Chem. Soc.*, **111**, 1299 (1989).
16. W. I. Sundquist, D. P. Bancroft and S. J. Lippard., *J. Am. Chem. Soc.*, **112**, 1590 (1990).
17. E. T. Martins, H. Baruah, J. Kramarczyk, G. Saluta, C. S. Day, G. L. Kucera and U. Bierbach, *J. Med. Chem.*, **44**, 4492 (2001).
18. L. Maresca, C. Pacifico, M. C. Pappadopoli and G. Natile, *Inorg. Chim. Acta.*, **304**, 274 (2000).
19. L. Monsù Scolaro, G. Alibrandi, R: Romeo and V. Ricevuto, *Inorg. Chem.*, **31**, 2074 (1992).
20. E. Ceci, R. Cini, J. Konopa, L. Maresca and G. Natile, *Inorg. Chem.*, **35**, 876 (1996).
21. Yu. N. Kukushkin, Yu. E. Vyaz'menskii, L. I. Zorina and Yu. L. Pazhukhina, *Russ. J. Inorg. Chem. (Engl. Transl.)*, **13**, 835 (1968).
22. M. J. Hynes, *J. Chem. Soc., Dalton Trans.* **1993**, 311.
23. R. Romeo, N. Nastasi, L. Monsù Scolaro, M. R. Plutino, A. Albinati and A. Macchioni, *Inorg. Chem.*, **37**, 5460 (1998).
24. S. Fortin and A. L. Beauchamp, *Inorg. Chem.*, **40**, 105 (2001).
25. A. Makriyannis, C. M. DiMeglio. A. P. DeJong and S. El-Khateeb, *Mol. Pharmacol.*, **43**, 78 (1998).
26. R. D. Green and J. S. Martin, *J. Am. Chem. Soc.*, **90**, 13659 (1968).
27. R. C. Jagessar, M. Shang, W. R. Scheidt and D. H. Burns, *J. Am. Chem. Soc.*, **120**, 11684 (1998)

